# Transcutaneous Electrical Acupoint Stimulation in Early Life Changes Synaptic Plasticity and Improves Symptoms in a Valproic Acid-Induced Rat Model of Autism

**DOI:** 10.1155/2020/8832694

**Published:** 2020-12-29

**Authors:** Xiaoxi Wang, Rui Ding, Yayue Song, Juan Wang, Chen Zhang, Songping Han, Jisheng Han, Rong Zhang

**Affiliations:** ^1^Department of Neurobiology, School of Basic Medical Sciences, Peking University, Beijing, China; ^2^Neuroscience Research Institute, Peking University, Beijing, China; ^3^Key laboratory for Neuroscience, Ministry of Education/National Health and Family Planning Commission, Peking University, Beijing, China; ^4^Department of Bioinformatics, School of Basic Medical Sciences, Peking University, Beijing, China; ^5^School of Basic Medical Sciences, Tianjin Medical University, Tianjin, China; ^6^Department of Neurobiology, School of Basic Medical Sciences, Beijing Key Laboratory of Neural Regeneration and Repair, Advanced Innovation Center for Human Brain Protection, Capital Medical University, Beijing, China; ^7^Wuxi HANS Health Medical Technology Co., Ltd., Wuxi, China; ^8^Department of Integration of Chinese and Western Medicine, School of Basic Medical Sciences, Peking University, Beijing 100191, China

## Abstract

Autism spectrum disorder (ASD) is a developmental disorder characterized by social behavior deficit in childhood without satisfactory medical intervention. Transcutaneous electrical acupoint stimulation (TEAS) is a noninvasive technique derived from acupuncture and has been shown to have similar therapeutic effects in many diseases. Valproic acid- (VPA-) induced ASD is a known model of ASD in rats. The therapeutic efficacy of TEAS was evaluated in the VPA model of ASD in the present study. The offspring of a VPA-treated rat received TEAS in the early life stage followed by a series of examinations conducted in their adolescence. The results show that following TEAS treatment in early life, the social and cognitive ability in adolescence of the offspring of a VPA rat were significantly improved. In addition, the abnormal pain threshold was significantly corrected. Additional studies demonstrated that the dendritic spine density of the primary sensory cortex was decreased with Golgi staining. Results of the transcriptomic study showed that expression of some transcription factors such as the neurotrophic factor were downregulated in the hypothalamus of the VPA model of ASD. The reduced gene expression was reversed following TEAS. These results suggest that TEAS in the early life stage may mitigate disorders of social and recognition ability and normalize the pain threshold of the ASD rat model. The mechanism involved may be related to improvement of synaptic plasticity.

## 1. Introduction

Autism spectrum disorder (ASD) is a developmental disorder. The core symptoms include social and communication deficits and stereotypic behavior. Together with its core symptoms, ASD patients also suffer from a lot of other cooccurring problems, especially hyperreactivity or hyporeactivity to sensory inputs and intellectual disability [1]. In the past few decades, the prevalence of ASD has increased dramatically. It is higher in boys than in girls, with an estimated ratio of 4 : 1 [2]. In DSM-V, the diagnostic criteria for perception appeared for the first time. As an earlier study reported, 90% of ASD children had an abnormal response to sensory stimulation [3]. Several studies have strongly implicated that an abnormal response to perception was one of the most obvious characteristics to distinguish ASD from other children with developmental disabilities [4–6]. Unfortunately, the etiology of ASD is still unclear, and the medical therapy is often one used to treat some associated symptoms, such as irritability and comorbidities [7]. Among the available methods for the treatment of ASD, rehabilitation training is most widely used for ASD intervention [7, 8]. But rehabilitation training often puts a heavy economic burden on families and society [9]. Besides, rehabilitation training has limitations, especially for infants with ASD [10].

The hypothesis for ASD pathogenesis mainly focuses on genetics and environment [11]. The molecular and cellular mechanisms mainly involve structural and functional abnormalities of the brain and synaptic plasticity abnormalities. Synapse generation and maturation is one of the key links in the development of brain neural circuits. In the early life stage, the number of dendritic spines increases rapidly. After reaching a peak, the density of dendritic spines in the brain stops increasing and gradually decreases, which is called “spine pruning” [12–14]. Spine pruning is considered to be related to neural circuit refinement which may be related to the pathogenesis of ASD [15]. In previous studies, dendritic spine density of ASD elder patients was significantly higher than that of the control [15–17].

Some evidence indicates that there is a relationship between the modulatory functions of the endocrine system and social behavior [18, 19]. As a part of the endocrine system, the hypothalamus can release a variety of neurotransmitters, such as oxytocin (OXT) and arginine-vasopressin (AVP). Because OXT and AVP were beneficial for regulating socio-emotional responses, they have attracted great interest for their critical implications for ASD [20–22]. Furthermore, the hypothalamus can also improve the emotional behavior by gut/axis and microbiota [23]. So, we pay more attention to hypothalamus in our current etiology study for ASD.

Acupuncture appears to be effective for treating many diseases and/or disorders by regulating the functions of the autonomic nervous system. Some research suggests that acupuncture could help ASD children to relieve their symptoms [24–26]. Moreover, meta-analysis results show that acupuncture could help ASD children reduce their Childhood Autism Rating Scale (CARS) and their Autism Behavior Checklist (ABC) score [27]. Transcutaneous electrical acupoint (TEAS) combines traditional acupuncture therapy with transcutaneous electrical nerve stimulation, which can produce effects similar to acupuncture [28].

Because TEAS is noninvasive stimulation, it is easier for children to accept. Consistent with our study, previous work in our lab also showed that TEAS would reduce part of ASD children's CARS and ABC total score, thereby improving their condition [29]. This previous study also demonstrated that TEAS simultaneously improved plasma levels of oxytocin and arginine-vasopressin [29]. Results in an animal study also done in our lab were similar to our clinical studies, showing that electro-acupuncture improved the social interaction behavior of rats [30].

However, few investigations have focused on the effect and mechanism of TEAS in early life. In this study, we use an autistic rat model to study the behavioral effect of TEAS in the early life stage and its influence on transcriptomics and synapse plasticity.

## 2. Material and Method

Male and female Wistar rats (270 g-350 g) were obtained from the Department of Experimental Animal Sciences, Peking University Health Science Center. Animals were housed individually with access to food and water. The humidity was 50% ± 10%, and temperature was 23 ± 2°C. The animals were maintained with a 12–12 h light-dark cycle. This study was carried out following USA National Institutes of Health Guide for the Care and Use of Laboratory Animals. The protocol was approved by Peking University Animal Care and Use Committee (ethics approval ID, LA2015204).

Female and male rats were placed in the same cage to mate overnight. The day was considered embryonic day 0.5 (E0.5) in the presence of a vaginal plug. The pregnant rats were randomly divided into two groups: VPA group and control (NS-control) group. In the VPA group, pregnant rats were intraperitoneally injected with VPA (Sigma: P4543, diluted with normal saline to a concentration of 200 mg/ml) at a dose of 450 mg/kg at embryonic day 12.5 (E12.5). The pregnant rats in the control group received the same concentration of normal saline at E12.5. After weaning at postnatal day 21 (PND21), offspring of the same sex were housed separately with 2-6 per cage.

**Figure 1:**
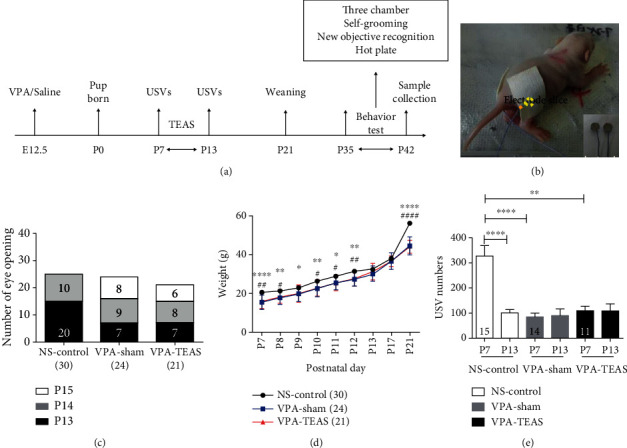
TEAS on PND7-PND13 did not improve the development and USVs of VPA-induced offspring. (a) Experimental design of TEAS intervention and behavior tests. (b) The photo is the offspring receiving TEAS. (c) The number of pups with eye-opened on certain days (two-way ANOVA, NS-control *n* = 30, VPA-sham *n* = 24, VPA-TEAS *n* = 21). (d) Body weight of pups from PND2 to PND21 (one-way ANOVA, NS-control *n* = 30, VPA-sham *n* = 24, VPA-TEAS *n* = 21). (e) Total USV numbers (PND7 vs. PND13: paired *t*-test, NS-control vs. VPA-sham vs. VPA-TEAS: one-way ANOVA, NS-control *n* = 15, VPA-sham *n* = 14, VPA-TEAS *n* = 11). Data are presented as the mean ± SEM. ^∗^*P* < 0.05, ^∗∗^*P* < 0.01, ^∗∗∗^*P* < 0.001; NS-control vs. VPA-TEAS, ^#^*P* < 0.05, ^##^*P* < 0.01.

On the postnatal day of 7th (PND7), the offspring of the VPA group were randomly divided into two groups: sham group (VPA-sham) and TEAS group (VPA-TEAS). The VPA-TEAS group was given transcutaneous electrical acupoint stimulation (JS-502-A manufactured at Wuxi HANS Health Medical Technology Company, Wuxi), with 6 mm electrodes attached on the acupoint of Zusanli (Figure 1(b)). TEAS duration was 7 days, from PND7 to PND13. Each day treatment duration was 30 minutes (Figure 1(a)). The current intensity of PND7 was 2 mA, PND8-10 was 3 mA, and PND11-13 was 4 mA. To be consistent with our previous clinical study, we chose 2/15 Hz as frequency (pulse width: 0.6 ms in 2 Hz and 0.4 ms in 15 Hz, each lasting for 3 s) [29].

The electrode was attached to the VPA-sham group on the same acupoint but without electrical stimulation. During stimulation, all of the offspring were placed in a clear plastic chamber, and a 37°C heating pad was placed under the box to maintain their body temperature.

### 2.1. Behavior Test

### 2.2. Developmental Milestone and Pup Separation-Induced Ultrasonic Vocalization Test

The developmental milestones of offspring were tested beginning from postnatal days 7 to 21(PND7-21). The parameters of physical developmental milestones included body weight and eye-opening. A pup separation-induced ultrasonic vocalization (USV) test was performed on postnatal days 7 (PND7) and 13 (PND13). A pup was randomly chosen and gently removed from the home cage and then transported to a clear plastic chamber (39 cm × 25 cm × 20 cm) on a heating pad (37°C) in a separate room. The USVs were recorded for 5 min by a condenser microphone (CM16/CMPA, Avisoft Bioacoustics, Germany) which hung 25 cm above the cage floor (AUSG-116H, Avisoft Bioacoustics, Germany). The sampling rate was set at 250 kHz. The connected amplifier (AUSG-116H, Avisoft Bioacoustics, Berlin, Germany) was set at a sampling rate of 250 kHz with a 125 kHz low-pass filter [31]. The USV data were analyzed by Avisoft SASLab Pro (Version 4.52).

#### 2.2.1. Three-Chamber Test

A three-chamber test was performed on postnatal days 35-42 (PND35–42) during the dark cycle [30]. The apparatus for testing was a rectangular Plexiglas box, which was divided into three chambers (40 cm × 34 cm × 24 cm), with the side chambers each connected to the middle chamber by a corridor (10 cm × 10 cm × 15 cm). The test included three stages: adaptive stage, stage 1 (social preference), and stage 2 (social novelty).

During the adaptive stage, the subject rat was allowed to explore the entire apparatus freely for 5 min. During stage 1, a weight and sex-matched unfamiliar model rat was retained in a small cage as a social stimulus and then placed in one of the side chambers. Then, an identical empty cage was placed on the other side of the chamber. The subject rat was allowed to explore the entire apparatus freely with the corridors open to allow interaction with the model rat and empty cage for 10 min. The social preference index was calculated as: (time in stranger side–time in empty cage side)/(time in stranger side + time in empty cage side) [32].

During stage 2, another weight and sex-matched unfamiliar model rat was placed in the empty cage as a novel social stimulus. The subject rat was also allowed to explore the entire apparatus and interact with the familiar and novel model rats for 10 min. The social novelty index was calculated as: (time in new stranger side–time in familiar stranger side)/(time in new stranger side + time in familiar stranger side). The entire apparatus was cleaned with 75% ethanol after each trial was completed to eliminate the impact of residual rat odors.

#### 2.2.2. Self-Grooming Test

The self-grooming test is a paradigm which measures the level of stereotyped behavior of rodents. During the dark period under dim red illumination, the subject rat was placed into an empty cage (39 cm × 25 cm × 20 cm), which was similar to the home cage, and encouraged to explore it for 10 min. In this study, self-grooming behaviors included (1) wiping the nose, face, head, and ears with forepaws and (2) licking the body, anogenital area, and tail [33]. The test consisted of a 10 min habituation and a following 10 min test. The test stage was videotaped, and the duration of self-grooming was analyzed.

#### 2.2.3. Novel Object Recognition Test

Learning and memory ability were evaluated by a novel object recognition test during the dark period under dim red illumination. A subject rat was placed in the test arena (60 cm × 40 cm × 40 cm) for 10 min of habituation on the first and second day. The training stage was on the third day. During the training stage, the rat was allowed to explore two identical objects in the arena for 20 min. 1 hour after training stage, the test stage started. One of the two objects would be replaced by a new object (with a similar size but with different colors and shapes). The rat was placed into the arena again to allow the animal to explore freely for 10 min and videotaped. The sniffing time for each of the two objects was determined by an observer blinded to the treatment group. Object exploration behavior was defined as the nose of the rat touching the object or being oriented toward the object within 2 cm.

#### 2.2.4. Hot Plate Test

The thermal nociception threshold was examined by a hot plate test. A solid aluminum plate was used for heating and maintaining a constant temperature. A Perspex cylinder which was transparent and removable was used. The temperature of the hot plate was set at 3 levels: 50°C, 53°C, and 56°C. After a rat was placed on the hot plate, the latent period was recorded for any of the behaviors: licking or lifting paws or jumping off the hot plate at each temperature. The paw withdrawal latency was intended to reflect the nociception threshold. A cut-off time of 60 s was set to avoid tissue damage. The subject rat was tested three times with 15 min intervals, and the mean of three recordings was the final result.

### 2.3. Golgi Staining

After the behavior tests, Golgi staining was used to evaluate neuron development of rats by the Hito Golgi-Cox OptimStainTM kit (HTKNS1125, Hitobiotec). Solution 1 and solution 2 of the kit were mixed in equal volumes at room temperature in a dark place 24 h before the experiment. The brain was removed and transferred into the mixed solution and remained for 2 weeks at room temperature in the dark. The brain tissue was transferred into solution 3 at 4°C for 24–72 h in the dark. After immersion in solution 3, the brain was frozen at 60°C. Coronal sections (150 mm) were prepared with a freezing microtome (Leica-1950, Germany). The sections were stained using solutions 4 and 5 after mounting the sections onto the slides with gelatin. Finally, the stained sections were imaged using a confocal microscope (TCS-SP8 STED 3X, Leica, Germany) equipped with a 40x oil immersion. Images were analyzed using the Fiji/Image J. For each rat, nine different neurons were quantified from three slides. The spine density from the three neuron segments (70 mm) was averaged to provide a single value for each type of neuron.

### 2.4. Transcriptomics

#### 2.4.1. Library Preparation for Transcriptome Sequencing

After behavior tests, the hypothalamus of the rat was collected for transcriptomics. A total amount of 1 *μ*g RNA per sample was used as input material for the RNA sample preparations. Sequencing libraries were generated using NEBNext® UltraTM RNA Library Prep Kit for Illumina® (NEB, USA) following the manufacturer's recommendations; index codes were added to attribute sequences to each sample. Briefly, mRNA was purified from total RNA using poly-T oligo-attached magnetic beads. Fragmentation was carried out using divalent cations under elevated temperature in NEBNext First Strand Synthesis Reaction Buffer (5x). First strand cDNA was synthesized using random hexamer primer and M-MuLV Reverse Transcriptase (RNase H-). Second strand cDNA synthesis was subsequently performed using DNA Polymerase I and RNase H. Remaining overhangs were converted into blunt ends via exonuclease/polymerase activities. After adenylation of 3′ ends of DNA fragments, the NEBNext Adaptor with hairpin loop structure was ligated to prepare for hybridization. In order to select cDNA fragments, preferentially of 250~300 bp in length, the library fragments were purified with the AMPure XPsystem (Beckman Coulter, Beverly, USA). Then, 3 *μ*l USER Enzyme (NEB, USA) was used with size-selected, adaptor-ligated cDNA at 37°C for 15 min followed by 5 min at 95°C before PCR. Then, PCR was performed with Fusion High-Fidelity DNA polymerase, Universal PCR primers, and Index (X) Primer. At last, PCR products were purified (AMPure XP system), and the quality was assessed on the Agilent Bioanalyzer 2100 system.

#### 2.4.2. Clustering and Sequencing

The clustering of the index-coded samples was performed on a cBot Cluster Generation System using TruSeq PE Cluster Kit v3-cBot-HS (Illumia) according to the manufacturer's instructions. After cluster generation, the library preparations were sequenced on an Illumina Nova seq platform, and 150 bp paired-end reads were generated.

#### 2.4.3. Differential Expression Analysis

Differential expression analysis of two conditions/groups (two biological replicates per condition) was performed using the DESeq2 R package (1.16.1). The DESeq2 provided statistical routines for determining differential expression in digital gene expression data using a model based on the negative binomial distribution. The resulting *P* values were adjusted using the Benjamin and Hochberg's approach for controlling the false discovery rate. Genes with *P* value < 0.05 found by DESeq2 were assigned as deferentially expressed.

#### 2.4.4. GO and KEGG Enrichment Analysis of Expressed Genes

The Rattus norvegicus PPI network was downloaded from the BioGRID database (https://thebiogrid.org/, v3.5.184). Gene Ontology (GO) enrichment analysis of differentially expressed genes and neighbor nodes were implemented by python package NetworkX [34]. GO terms with *P* value less than 0.05 were considered significantly enriched by differentially expressed genes.

KEGG is a database resource for understanding high-level functions and utilities of a biological system, such as the cell, the organism, and the ecosystem. It is based on molecular-level information, especially large-scale molecular datasets generated by genome sequencing and other high-throughput experimental technologies (https://www.genome.jp/kegg/). We used DAVID v6.8 (https://david.ncifcrf.gov/) to test the statistical enrichment of differential expression genes in KEGG pathways [35, 36].

### 2.5. Statistics

IBM SPSS Statistics 19 (SPSS Inc., Chicago, IL, USA) and GraphPad Prism 5.0 (GraphPad Software Inc., San Diego, CA, USA) were used for statistical analyses and generating graphs. For the comparisons, parametric tests including *t*-tests and one-way analysis of variance (ANOVA) were used if the data was normally distributed (distribution tested by the Shapiro-Wilk normality test), and nonparametric approaches, including the Wilcoxon test and Kruskal-Wallis test, were used for data with a nonnormal distribution. Pearson's chi-squared test was used to assess rank variables. For all data, the results were expressed as the mean ± standard error of the mean (SEM), and *P* < 0.05 (two-tailed) was considered statistically significant.

## 3. Result

### 3.1. TEAS Effect on Development Milestone and USVs

To evaluate the therapeutic effect, the TEAS treatment was administered from PND7 to PND13. There were no statistical differences in eye-opening time among the NS-control group, VPA-sham group, and VPA-TEAS group (two-way ANOVA, Figure 1(c)). The body weight of the NS-control group was higher than the VPA group (one-way ANOVA, *P* < 0.05, Figure 1(d)). The total number of USVs was lower in the VPA-sham and the VPA-TEAS group compared with that in the NS-control group on PND7. And the USV number was significantly lower in the NS-control group on PND13 compared with that on PND7 (paired *t*-test, *P* < 0.05, Figure 1(e)). But there were no differences between the VPA-sham and VPA-TEAS group on PND7 or PND13 (Figure 1(e)). These results showed that TEAS in early life did not improve the development and communication ability of the VPA-induced offspring.

### 3.2. Early TEAS Produced a Long-Term Behavioral Effect in Adolescence

Sociability, self-grooming, and cognition ability were evaluated during adolescence.

**Figure 2:**
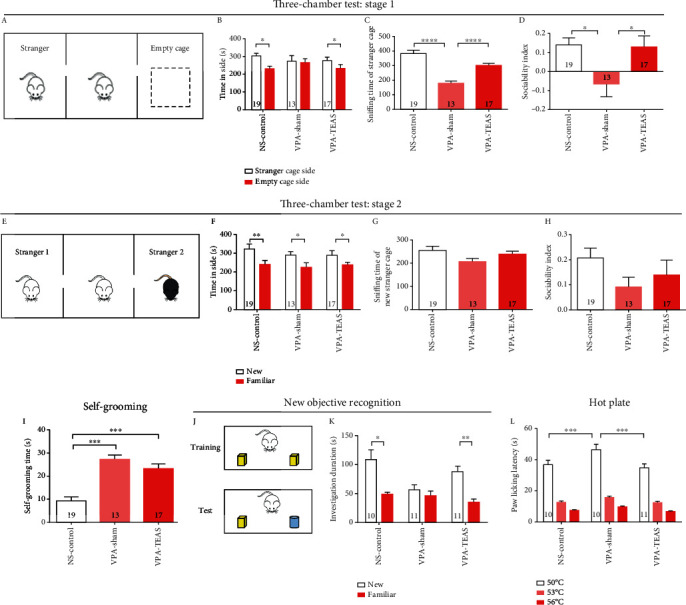
TEAS treatment in early life stage had long-term behavior effects on offspring of VPA treated rats. (a) Schematic diagram of three-chamber test in stage 1 (social preference). (b) The time the animal spent investigating either on the stranger cage side or empty cage side in stage 1 of the three-chamber test (paired *t*-test, NS-control *n* = 19, VPA-sham *n* = 13, VPA-TEAS *n* = 17). (c) The sniffing time of the stranger cage in stage 1 of the three-chamber test (one-way ANOVA). (d) The social preference index (one-way ANOVA). (e) Schematic diagram of the three-chamber test in stage 2 (social novelty). (f) The time spent investigating either the new stranger or the familiar stranger in stage 1 of the three-chamber test (paired *t*-test, NS-control *n* = 19, VPA-sham *n* = 13, VPA-TEAS *n* = 17). (g) The sniffing time on new stranger in stage 2 of the three-chamber test (one-way ANOVA). (h) The social preference index (one-way ANOVA). (i) The time of self-grooming test (one-way ANOVA, NS-control *n* = 19, VPA-sham *n* = 13, VPA-TEAS *n* = 17). (j) Schematic diagram of new objective recognition. (k) The time spent on investigating either familiar or novel object in novel object recognition test (one-way ANOVA, NS-control *n* = 10, VPA-sham *n* = 10, VPA-TEAS *n* = 11). **(**l) The withdrawal latency in the hot plate test for heat sensitivity (one-way ANOVA for same temperature, NS-control *n* = 10, VPA-sham *n* = 10, VPA-TEAS *n* = 11). Data was presented as the mean ± SEM. ^∗^*P* < 0.05, ^∗∗^*P* < 0.01, ^∗∗∗^*P* < 0.001.

1. *Social preference*: in stage 1 of the three-chamber tests, the NS-control group and the VPA-TEAS group spent more time on the stranger rat side than on the empty cage side (paired *t-*test, *P* < 0.05, Figure 2(b)). On the contrary, there was no difference between the stranger rat side and the empty cage side of the VPA-sham group (paired *t*-test, *P* = 0.95, Figure 2(b)). The sniffing time of the stranger rat in the VPA-sham group was lower than that in the NS-control group and the VPA-TEAS group (one-way ANOVA, *P* < 0.001, Figure 2(c)). The result in the social index was consistent with sniffing time, which for the VPA-sham group was lower than for the NS-control group and the VPA-TEAS group (one-way ANOVA, *P* < 0.05, Figure 2(d)). These results suggested that offspring of the VPA-treated rat had social preference deficits, and early TEAS intervention would repair the social preference deficits in this VPA-induced rat model of ASD
2. *Social novelty*: in stage 2 of the three-chamber tests, all of the three groups (NS-control group, VPA-sham group, and VPA-TEAS group) showed a preference for the new stranger rat (paired *t*-test, *P* < 0.05, Figure 2(f)). No differences in sniffing time of the new stranger rat or the social index of social memory were observed among the three groups
3. *Repetitive behavior*: repetitive behavior manifested as self-grooming behavior was analyzed. The results suggested that the VPA-sham and the VPA-TEAS group had more repetitive behavior (one-way ANOVA, *P* < 0.001, Figure 2(i))
4. *Cognitive ability*: cognitive ability was examined with a novel object recognition test. During the test stage, the NS-control and the VPA-TEAS group spent more time sniffing the new object than the VPA-sham group (paired *t*-test, *P* < 0.05, Figure 2(k)). This result indicated that early TEAS would improve the cognitive ability of the VPA rat in adolescence
5. *Hot plate*: in the hot-plate experiment, the data showed that paw withdrawal latency increased significantly for the VPA-sham group compared with the NS-control and VPA-TEAS group at 50°C (one-way ANOVA, *P* < 0.001, Figure 2(l)). At 53°C and 56°C, there was no difference in the paw withdrawal latency among the three groups. This result suggested that pain perception impaired in this VPA rat model of ASD and TEAS intervention in the early stage would mitigate the abnormal pain perception in adolescence

### 3.3. Improvement by Early TEAS on Dendritic Spine Pruning in S1

**Figure 3:**
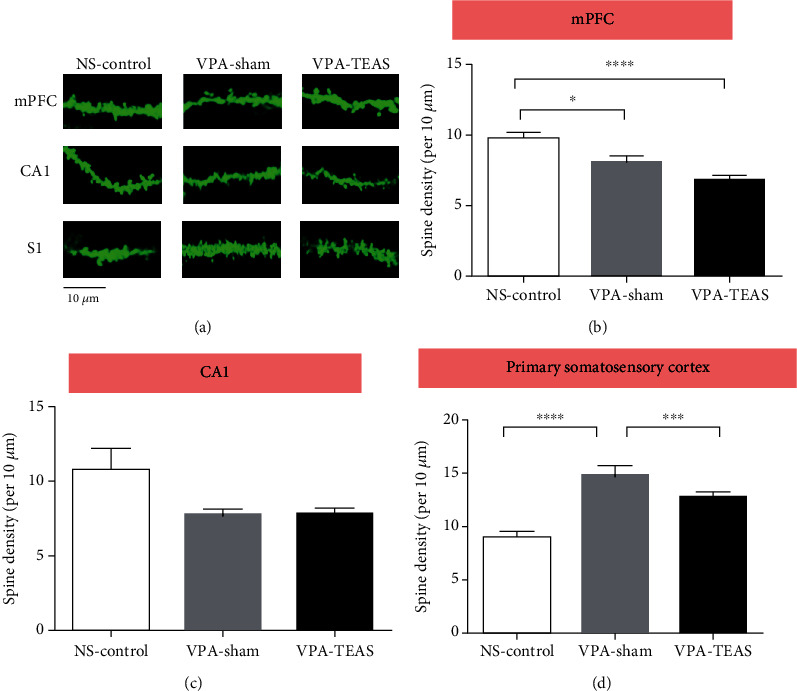
TEAS in early life would repair the deficit of spine pruning on VPA rats in S1 neurons. **(**a) Representative images of dendritic spines from mPFC, CA1, and S1 neurons (scale bar = 10 *μ*m). **(**b) The spine density of mPFC in PND42 (one-way ANOVA). (c) The spine density of CA1 in PND42 (one-way ANOVA). **(**d) The spine density of primary somatosensory cortex (S1) in PND42 (one-way ANOVA, *n* = 4 rats for each group, nine neurons per rat). Data was presented as mean ± SEM. ^∗^*P* < 0.05, ^∗∗∗^*P* < 0.001, ^∗∗∗∗^*P* < 0.0001.

Dendritic spine density in medial prefrontal cortex (mPFC), hippocampus CA1, and primary somatosensory cortex (S1) were analyzed by Golgi staining after behavior tests. In mPFC, spine density of the VPA-sham and VPA-TEAS groups was lower than that of the NS-control group (one-way ANOVA, *P* < 0.05, Figure 3(b)). In CA1, a tendency towards a lower spine density was seen in the VPA-sham and VPA-TEAS groups (one-way ANOVA, *P* = 0.09, Figure 3(c)). Interestingly, the spine density was increased in the VPA-sham group compared with the NS-control in PND42. The increased spine density was partially reversed in the VPA-TEAS group (one-way ANOVA, *P* < 0.001, Figure 3(d)). This finding indicated that early TEAS would be beneficial for facilitating spine pruning in certain brain regions.

### 3.4. Transcriptome Analysis

The hypothalamus is located under the cerebral cortex that can release a variety of neurotransmitters. Hypothalamic neurons have extensive synaptic connections with nerve fibers from other parts. To detect the synaptic changes produced by TEAS, we chose transcriptome to analyze the hypothalamus changes.

#### 3.4.1. Differential Gene Analysis

**Figure 4:**
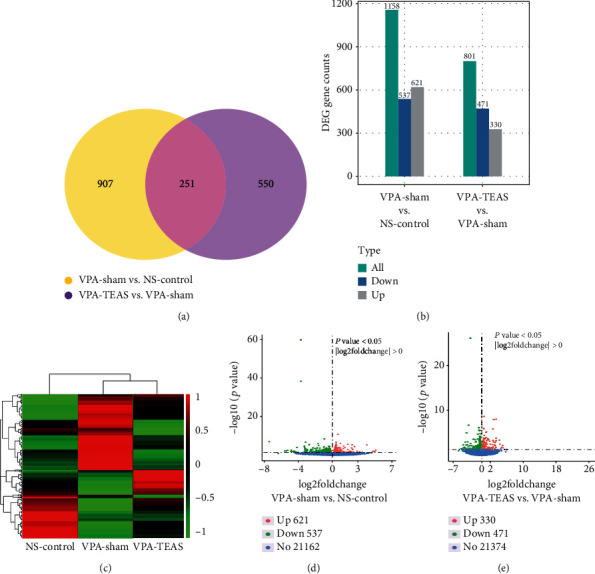
Differential gene analysis of the hypothalamus. (a) The Venn chart of differential gene among NS-control, VPA-sham, and VPA-TEAS group (*n* = 3 for each group). (b) Distribution of differential genes of NS-control, VPA-sham, and VPA-TEAS group. (c) Clustering of NS-control, VPA-sham, and VPA-TEAS group in heat map diagram. (d) Volcano plot of differential genes between VPA-sham and NS-control. (e) Volcano plot of differential genes between VPA-TEAS and VPA-sham.

Transcriptome referred to the total of all RNA transcribed by a specific tissue or cell at a certain time or state. Differential gene analysis revealed that compared with the NS-control group, the VPA-sham group had 1158 genes with changed expression patterns, including 621 upregulated genes and 537 downregulated genes (Figures 4(a), 4(b), and 4(d)). Furthermore, the VPA-TEAS group had 801 genes changed in comparison with the VPA-sham group, including 330 genes upregulated and 471 genes downregulated (Figures 4(a), 4(b), and 4(e)). As shown in Figure 4(a), 251 changed genes were commonly found among three groups.

#### 3.4.2. GO Analysis

**Figure 5:**
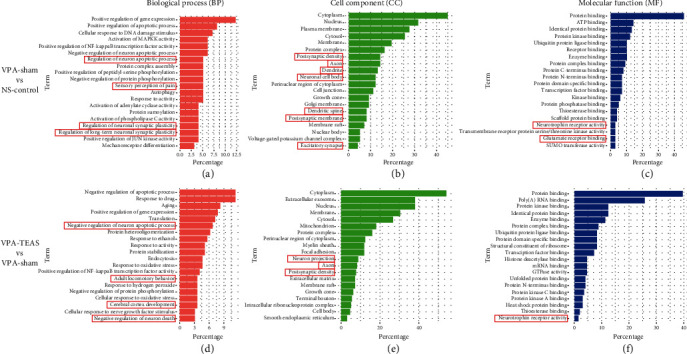
GO analysis of differential terms in BP, CC, and MF terms: (a–c) downregulated terms in BP, CC, and MF frequency between the VPA-sham and the NS-control; (d–f) upregulated terms in BP, CC, and MF frequency between the VPA-TEAS and the VPA-sham. (The red color presents biological process terms. The green color presents cell component terms. The blue color presents molecular function terms.)

GO analysis included the biological process (BP), cell component (CC), and molecular function (MF). In BP terms (Figure 5(a)), the autism-related GO terms were regulation of neuron apoptotic process (GO:0043523), sensory perception of pain (GO:0019233), regulation of neuronal synaptic plasticity (GO:0048168), and regulation of long-term neuronal synaptic plasticity (GO:0048169). In CC terms (Figure 5(b)), autism-related GO terms were postsynaptic density (GO:0014069), axon (GO:0030424), dendritic spine (GO:0043197), neuronal cell body (GO:0043025), postsynaptic membrane (GO:0045211), and excitatory synapse (GO:0060076). In MF terms (Figure 5(c)), the autism-related GO terms are neurotrophin receptor binding (GO:0005168) and glutamate receptor binding (GO:0035254). These findings suggested that the VPA-induced rat model would experience autism-like behavior through synaptic dysfunction.

Between the VPA-TEAS and the VPA-sham group in BP terms (Figure 5(d)), biological process terms which related to autism were negative regulation of neuron apoptotic process (GO:0043524), cerebral cortex development (GO:0021987), and negative regulation of neuron death (GO:1901215). In CC terms (Figure 5(e)), autism-related GO terms were neuron projection (GO:0043005), axon (GO:0030424), and postsynaptic density (GO:0014069). In MF terms (Figure 5(f)), the autism-related GO term was neurotrophin receptor activity (GO:0005030). These results showed that early TEAS would improve neuron development of the VPA rats.

#### 3.4.3. KEGG

**Figure 6:**
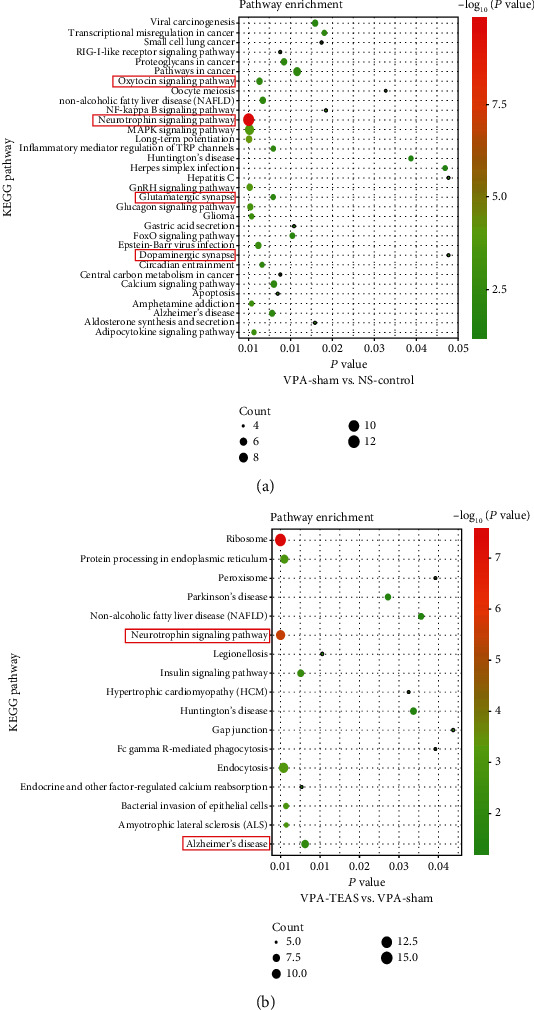
The Kyoto Encyclopedia of Genes and Genomes (KEGG) pathways enrichment: (a) KEGG enrichment pathway of downregulation in the VPA-sham compared with the NS-control; (b) KEGG enrichment pathway of upregulated in the VPA-TEAS compared with VPA-sham. (The black points present the number of enriched genes. The color from red to green presents *P* value of pathway analysis).

KEGG pathway analysis of the integrated differentially expressed genes was performed using the DAVID database, and the results of the analysis are shown in Figure 6. Compared with the NS-control group, the downregulated differentially expressed genes were mainly enriched in the oxytocin pathway, neurotrophic signaling pathway, glutamatergic synapse, and dopaminergic synapse, which were related to the ASD pathogen (Figure 6(a)). And then, we found that the upregulated differentially expressed genes of the VPA-TEAS group were mainly enriched in the neurotrophic signaling pathway (Figure 6(b)). Thus, the neurotrophic signaling pathway would be a potential target for ASD intervention.

## 4. Discussion

Our findings showed that the VPA-induced rat model had impaired social, cognition, and sensory functions. Moreover, TEAS in early life could repair the above deficits in adolescence. Finally, early TEAS would be beneficial to spine pruning and neuron development of the VPA-induced rat model.

Epidemiology demonstrated that the offspring of mothers taking VPA during pregnancy would be three times more likely to suffer ASD than those not taking VPA [37]. So VPA is commonly used in the autism-like animal model. In our study, prenatal exposure to VPA was found to lead to social, cognition, and sensory impairment of offspring, which was consistent with previous research [38].

In the previous ASD animal studies, the time of acupuncture intervention was usually selected after PND21 [39–41], but in our study, we chose PND7 to PND13 as the TEAS time. That was because PND7 to PND13 was about 1-2 years old in humans. Firstly, we chose this period to be consistent with our previous clinic study [29]. Secondly, TEAS had a better effect in 3-6 years old ASD children than those above 7 [37]. More importantly, according to the Centers for Disease Control and Prevention (CDC) recommendation, early intervention can significantly improve the development of ASD children [7]. Treatment for ASD children should be started as soon as possible, when ASD symptoms appear. The earliest appearance of ASD symptoms is at 18-24 months after birth [42]. This period met the CDC's recommendation for the early intervention stage of ASD.

In parameters selection, we used 2/15 Hz as the frequency, which is the same one used in our clinical study. And 2/15 Hz TEAS was also effective for treating the ASD children according to our previous work [29]. In this study, we observed that early TEAS would repair the deficits of social, cognition, and abnormal heat sensation. As a noninvasive stimulus, TEAS would be more suitable for young animals than manual or electrical acupuncture.

Several lines of evidence from our study suggested that the etiology of ASD was related to genetic and environmental risk factors [1, 11] which would affect synaptic function and neuron development. The density of dendritic spines changed dynamically, increasing in early life and starting to decrease in adolescence. And this kind of dendritic spine change has been called spine pruning, which is related to precision functioning of the central nervous system [38, 43]. The change of spine pruning was roughly the same in different brain regions, but time and extent were different [14, 44].

Acupuncture has been practiced in China for over 2000 years to treat a variety of diseases [45]. The methods of acupuncture were integrated into electrical acupuncture and TEAS. Physiological effects of TEAS are similar to manual acupuncture in analgesia, etc. [46]. Cumulative evidences have demonstrated that acupuncture could induce neural plasticity in rodents [47]. And our Golgi staining results showed that TEAS would decrease the spine density of the VPA rat model in S1 at adolescence.

But we did not observe the same phenomenon in mPFC and CA1. In the previous studies, acupuncture has been found to play a positive role in improving the expression levels of synaptophysin and PSD-95 [48, 49]. Most of the brain area acupuncture research is concerned with the hippocampus and cortex [50–53]. For example, in the previous study, it was found that acupuncture modulated the excitability of the motor cortex, and the plasticity was time-dependent [54]. Furthermore, it was suggested that the synaptic effect may be due to the changes in the synaptic structure of the brain, the detection time, and the acupoint [47]. Our results may give a hint that TEAS may have brain region specificity in the ASD animal model. Simultaneously, the synaptic effect of TEAS, such as spine pruning, may be distinct in different life periods. Moreover, it is plausible that TEAS in early life might repair the deficit of spine pruning at adolescence in the VPA rat model, which would help the ASD animal model to repair abnormal neural circuits. In the future study, we will seek related molecular factors to analyze the TEAS synaptic effect in different brain areas and at different life times.

The synaptic function changes are accompanied by molecular changes. Because of the difficulty of brain tissue collection, there is limited data on transcriptomics of ASD patients. The prior work has suggested that there was dysregulation of the AMPA receptor subunit expression in the cerebellum of ASD patients [55]. Meanwhile, ASD patient brain research found that the gene expression of the immune response was upregulated, while that involved in the synaptic function was downregulated [56]. In an ASD animal study, many genes and proteins were found to be changed, such as the autism-related susceptibility genes BDNF, Shank3, and ERK1 [57]. In the study of the VPA mouse model, Neu2 and Mt2a had a significant decrease in the amygdala [58]. Besides, transcriptomics found that the impairment in the VPA model in the brain area involved the orbital frontal lobe and cerebellar vermis [59].

But there was little information about the hypothalamus of ASD transcriptomics in previous studies. This study indicated that some genes significantly changed in the VPA rat model, like the genes postsynaptic density, the excitatory synapse, and sensory perception of pain. Moreover, our present KEGG study showed that the pathways related to ASD in the VPA rat model included the oxytocin signaling pathway, the neurotrophic signaling pathway, the glutamatergic synapse, and the dopaminergic synapse. These pathways were related to the synapse, indicating that VPA was associated with the occurrence and development of synapses in offspring.

Transcriptomic studies provide only limited information regarding medical treatment of ASD at this time. The possible targeted intervention goals from other previous studies included synapse function, chromatin modification and transcriptional regulation, neuron projection, and neurogenesis [60, 61]. Our findings indicated that early TEAS will improve the postsynaptic density, axon, and neuron projection of the VPA rat model. These results were related to the pathogenesis of ASD and might provide potential targets for treatment.

Nerve growth factor is the earliest discovered neurotrophic factor and can provide nutrition for neurons and induce neurite outgrowth [62]. It was reported that the neurotrophic factor level of an ASD rat was lower than that of the control [63, 64]. There was also a report that spine maturation required the neurotrophic factor [65]. Previously, other studies reported that acupuncture or electrical acupuncture would increase the expression of the neurotrophic factor [66–68]. In line with these results, we could infer that TEAS would promote spine maturation by increasing the expression of neurotrophic factors.

A possible limitation of our study is the potential relation between spine maturation and transcript factor which should be explored more deeply. And our transcriptomic results showed that early TEAS would not only improve the synapse function but also other pathways, such as ribosome and peroxisome. In the future, we hope to find more evidence to support the therapeutic effect of TEAS on ASD.

## 5. Conclusions

The present study demonstrates that a VPA-induced rat will have autistic like behavior deficits. The reason for the behavior deficits is related to spine pruning and synaptic impairment. Equally important, TEAS in early life can repair the social, cognition, and heat insensitivity impairments and have a long-term positive effect on the synapse function in adolescence.

## Data Availability

The data used to support the findings of this study are available from the corresponding author upon request.
